# Antiplatelet and Antithrombin Strategies in Acute Coronary Syndrome: State-Of-The-Art Review

**DOI:** 10.2174/157340312803217193

**Published:** 2012-08

**Authors:** Refai Showkathali, Arun Natarajan

**Affiliations:** Specialist Registrars in Cardiology, The Essex Cardiothoracic Centre, Essex, United Kingdom

**Keywords:** Acute coronary syndrome, ACS, antiplatelet agents, antithrombotic agents, bivalirudin, cangrelor, NSTEMI, STEMI.

## Abstract

Antiplatelet and antithrombotic agents significantly alter the clinical course of patients with acute coronary syndrome (ACS) and hence form the bedrock of the management pathway of this closely related continuum of coronary pathologies. The contemporary therapeutic armamentarium for the treatment of ACS now reflects the many technical and pharmacological advances that took place over the last two decades. In the original 1996 American College of Cardiology/American Heart Association (ACC/AHA) guidelines for the management of acute myocardial infarction, only one antiplatelet agent (Aspirin) and one anticoagulant (unfractionated heparin) were recommended as class I therapies. Since then many newer agents have been developed and approved for routine clinical use in ACS patients. Recent research has focussed on improving efficacy on one hand and reducing bleeding complications on the other. This review focuses on the mechanism, efficacy, safety profile and clinical trial evidence of P2 Y_12_ receptor antagonist antiplatelet agents, glycoprotein IIb/IIIa receptor inhibitors (GPI), protease-activated receptor-1 (PAR-1) inhibitors, thrombin inhibitor bivalirudin and Factor Xa inhibitors fondaparinaux and rivaroxaban.

## INTRODUCTION

**Fig. (1):**
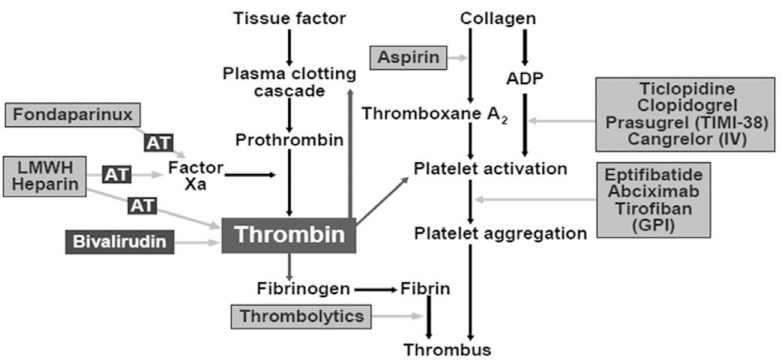
Role of thrombin in the process of tissue injury, coagulation and platelet response is shown along with the site of action of drugs. AT = antithrombin III, ADP = adenosine diphosphate, LMWH heparin = low molecular weight heparin, TIMI-38 = Thrombolysis In Myocardial Infarction-38 study, IV = intravenous.

Acute coronary syndromes (ACS), which include ST-segment elevation myocardial infarction (STEMI), non-ST-segment elevation myocardial infarction (NSTEMI) and unstable angina (UA), present a unique challenge to clinicians because of the high rate of mortality and morbidity associated with these conditions. ACS occurs after the rupture of an inflamed atherosclerotic plaque, which exposes prothrombotic contents of the vascular matrix to flowing blood. Following plaque rupture, circulating platelets adhere to, and are activated by the exposed components of the vascular matrix. Tissue factor is exposed to plasma after plaque rupture, initiating the thrombotic cascade *via* its interaction with coagulation factor VII. Activated factor VII (factor VIIa) molecules activate small amounts of factor X that combine with factor Va to produce thrombin (factor IIa) (Fig. **1**). Thrombin (*vide infra*) then serves to amplify the response to the injury by further activation of platelets. These interactions result in the assembly of the prothrombinase complex on the surface of activated platelets and the generation of large amounts of thrombin that catalyzes the production of fibrin and cause the clinical manifestations of thrombosis and ACS [1]. As a result, the treatment of ACS typically includes the use of antiplatelet and antithrombotic agents, in addition to percutaneous coronary intervention (PCI) or surgical revascularisation. In the original 1996 American College of Cardiology/American Heart Association (ACC/AHA) guidelines for the management of acute myocardial infarction, only one antiplatelet agent (Aspirin) and one anticoagulant (unfractionated heparin) were recommended as class I therapies. Since then many newer agents have been developed and approved for routine clinical use in ACS. 

The consummate antiplatelet agent would be required to have a rapid onset of action, deliver complete platelet blockade, be fully reversible and provide optimal anti-ischaemic effects in the absence of an increase in bleeding risk. Research over the last two decades has delivered drugs which have varying levels and combinations of the above named features and act synergistically with aspirin. This review focuses on the mechanism, efficacy, safety profile and clinical trial evidence of a) the P2Y_12_ receptor antagonists clopidogrel, prasugrel, ticagrelor and cangrelor, b) platelet glycoprotein IIb/IIIa receptor inhibitors (GPI), c) thrombin inhibitor bivalirudin and d) Factor Xa (FXa) antagonists- subcutaneous fondaparinux and oral rivaroxaban. The review is confined to those agents that have been tested in large phase 3 clinical trials and many of these are in current clinical use.

## PLATELET P2Y_12_ RECEPTOR ANTOGONISTS

### Clopidogrel

Clopidogrel is the most widely used thienopyridine derivative, after ticlopidine was sidelined because of side effects such as neutropenia [2]. Clopidogrel induces irreversible alterations of the platelet receptor P2Y_12_ mediating inhibition of stimulated adenylyl cyclase activity by adenosine diphophate (ADP) [3, 4]. It inhibits platelet aggregation by a mechanism different to that of aspirin and thus adds to its effects. In one of the earliest trials of clopidogrel that involved patients with stable atherosclerosis, it was found to be superior to aspirin with a comparable safety profile [5]. Its use in combination with aspirin in patients with NSTEMI was established by the landmark Clopidogrel in Unstable Angina to Prevent Recurrent Events (CURE) trial [6]. In this study ischaemic cardiovascular events or cardiovascular death occurred in 16.5% of the patients treated with clopidogrel and aspirin therapy compared to 18.8% of the patients treated with aspirin alone, over a 3-12 months period (RR, 0.86; 95% CI, 0.79 to 0.94; P<0.001). The benefit of dual antiplatelet therapy during PCI and for up to one year thereafter was demonstrated in subsequently published randomised studies [7, 8]. The Chinese Clopidogrel and Metoprolol in Myocardial Infarction Trial (COMMIT) trial [9] and another American study [10], both published in 2005 demonstrated superiority of clopidogrel and aspirin combination over aspirin alone in patients with STEMI. 

Clopidogrel is now routinely used in patients with all forms of acute coronary syndrome (ACS) including those undergoing primary PCI. But it has a few drawbacks. It is a prodrug and the two-step activation process, involving a series of cytochrome P-450 (CYP) isoenzymes is susceptible to the interference of genetic polymorphisms and drug interactions [11]. Accordingly, clopidogrel a) has a delayed onset of action, b) causes irreversible platelet blockade and b) has large interindividual variability in platelet response which is sometimes termed as “clopidogrel resistance” [12]. Newer P2Y12 antagonists have now been developed and tested in phase III trials and are summarized below.

#### Clopidogrel and proton pump inhibitors:

There is one other potential area of concern with clopidogrel. Some observational studies have suggested that there may be an interaction between clopidogrel and proton pump inhibitors (PPIs) that, if real, could have blunting of its antiplatelet efficacy and ensuing clinical effects [13, 14]. These studies have been bolstered by results of *ex vivo* analyses, many of which have shown inhibition of the antiplatelet effect of clopidogrel by PPIs, most consistently omeprazole [15-17]. A number of other observational studies, however, did not show an interaction between clopidogrel and PPIs [18, 19]. A recent randomised controlled study to assess the efficacy and safety of concomitant administration of clopidogrel and omeprazole (as a combination pill) in patients with coronary artery disease who received clopidogrel plus aspirin did not show any significant difference in cardiovascular events in the omeprazole arm when compared to placebo (4.9% vs 5.7%, hazard ratio [HR] 0.99; 95% confidence interval [CI], 0.68 to 1.44; P=0.96). A favourable difference in gastrointestinal (GI) outcomes was evident with the addition of a PPI to clopidogrel; there was a 45% relative risk reduction (RRR) for GI bleeding events [20]. It is important to note that the combination pill contained 75 mg clopidogrel around a core of delayed-release omeprazole. This is quite important in clinical practice as this combination separated the absorption of clopidogrel from that of the PPI and may have significantly reduced the competitive inhibition of the enzyme CYP2C19 by omeprazole. On balance, the Committee on Human Medicinal Products (CHMP) discourage the concomitant use of clopidogrel and omeprazole or esomeprazole in clinical practice [21]. In patients who do require PPIs, use of pantoprazole in place of omeprazole or lansoprazole is recommended. This is due to the fact that pantoprazole does not appear to have as significant an inhibitory effect on the cytochrome enzyme CYP2C19 as other PPIs.

### Prasugrel

Prasugrel is a novel thienopyridine which binds to the platelet P2Y_12_ receptor to confer antiplatelet activity. It is a prodrug and like clopidogrel, requires conversion to an active metabolite before being able to exert antiplatelet action. However, prasugrel inhibits ADP–induced platelet aggregation more rapidly, more consistently, and to a greater extent compared to clopidogrel in patients with ACS [22, 23], including those undergoing PCI [23]. Indeed, pharmacodynamic data have shown that the degree of inhibition of platelet aggregation achieved with prasugrel within 30 minutes after treatment is similar to the peak effect of clopidogrel that is seen 6 hours after administration [24].

Clinical outcomes evidence for prasugrel comes from the Therapeutic Outcomes by Optimizing Platelet Inhibition with Prasugrel–Thrombolysis in Myocardial Infarction (TRITON–TIMI) 38 trial [25]. This was a randomised double-blinded trial that compared prasugrel with clopidogrel in 13,608 moderate to high-risk ACS patients who were scheduled to have PCI. All patients were given aspirin and randomised to receive a loading dose of 60 mg prasugrel followed by 10 mg prasugrel daily or a loading dose of 300 mg clopidogrel followed by 75 mg clopidogrel daily for up to 15 months. The intention-to-treat analysis showed that the primary efficacy endpoint, a composite of non-fatal myocardial infarction (MI), non-fatal stroke or death from cardiovascular causes, was reached in 9.9% of patients in the prasugrel group and 12.1% of patients in the clopidogrel group (HR 0.81; 95% CI 0.73 to 0.90; P<0.001). This equated to a 2.2% absolute risk reduction (ARR) and a 19% RRR in the primary endpoint. The rates of individual ischemic events were also reduced in the prasugrel group, with a 2.3% ARR and a 24% RRR for myocardial infarction, a 1.2% ARR and a 34% RRR for urgent target-vessel revascularization, and a 1.3% ARR and a 52% RRR for stent thrombosis, a rare but catastrophic event. There was no demonstrable reduction in mortality with prasugrel [25].

The problem with prasugrel however, was the increased bleeding risk. Both Thrombolysis in myocardial infarction (TIMI) non-coronary artery bypass grafting (non-CABG) major bleeding (fall in haemoglobin of 5 g/100 ml) and life-threatening bleeding were increased with prasugrel compared to clopidogrel (2.4% versus 1.8%, HR 1.32; 95% CI 1.03 to 1.68, P=0.03 and 1.4% versus 0.9%, HR 1.52; 95% CI 1.08 to 2.13; P=0.01).The rate of coronary artery bypass graft surgery (CABG)-related bleeding was also increased with prasugrel although the numbers were low. To put this in perspective, the estimated number of patients needing treatment with prasugrel, as compared with standard-dose clopidogrel, to prevent one primary efficacy end point during a 15-month period was 46. The number of patients who would have to be treated to result in an excess non–CABG-related TIMI major bleed was 167. Accordingly, prasugrel must be avoided in the elderly aged ≥75 years, those weighing <60 kg, patients with previous strokes and in those due to undergo urgent CABG [25].

A potential flaw with the design of TRITON-TIMI 38 was that patients were given prasugrel or clopidogrel only after the coronary anatomy was known (following angiography), which could have disadvantaged clopidogrel. Indeed, a large proportion of the benefit seen in the study was early. In addition, clopidogrel loading was performed with a lower dose (300 mg). Many centres including ours in the UK now use 600 mg loading and furthermore, this loading is done on diagnosis of ACS, before coronary anatomy is determined. But given the positive efficacy result from the trial, it may be argued that pre-treatment is not be required with prasugrel given its relatively rapid onset of action and may therefore be an advantage.

The US Food and Drugs Administration (FDA) approved prasugrel for use during PCI in the setting of ACS in July 2009. The UK National Institute of Health and Clinical Excellence (NICE) approved the use of prasugrel in October 2009 in those with ACS undergoing PCI who have STEMI, stent thrombosis whilst taking clopidogrel or diabetes mellitus [26].

### Ticagrelor

Ticagrelor is a reversible and direct-acting oral P2Y_12_ antagonist and provides faster, greater, and more consistent antiplatelet action than clopidogrel [27, 28]. A head-to-head comparison of ticagrelor and clopidogrel was made in the large PLATelet inhibition and patient Outcomes (PLATO) trial [29]. In this study over 18,000 patients admitted with ACS, were randomised to receive ticagrelor or clopidogrel, both with loading doses. The primary composite end point occurred less frequently in patients receiving ticagrelor (9.8%) as compared with those receiving clopidogrel (11.7%) (HR, 0.84; CI, 0.77 to 0.92; P<0.001). Secondary end points of MI and death from vascular causes also occurred less frequently in the ticagrelor group. These benefits were observed irrespective of whether a higher loading dose of clopidogrel was used and a non-invasive rather than invasive strategy was adopted. Although there was no increase in the rates of major bleeding with ticagrelor as defined in the trial or by TIMI criteria, there was a higher incidence of non-CABG bleeding (4.5% vs. 3.8%, P = 0.03), including more instances of fatal intracranial bleeding. In addition, two other problems were seen more frequently in the ticagrelor arm - dyspnoea and a higher incidence of ventricular pauses on holter monitoring although the pauses were rarely associated with symptoms [29].

Crucially, there was significant reduction in mortality from use of ticagrelor - death due to vascular causes was 4.0% vs. 5.1%, (P=0.001) and death from any cause 4.5%, vs. 5.9% (P<0.001). This translated into an ARR of 1.4% and RRR of 22% in the rate of death from any cause at 1 year with use of ticagrelor [29]. In contrast, other contemporary antiplatelet trials involving clopidogrel [6], prasugrel [25], or glycoprotein IIb/IIIa inhibitors (GPI) [30] in patients with ACS have not shown mortality reduction. 

There has however been criticism aimed at the trial data showing mortality reduction. The North American centres in that the trial showed a paradoxical trend towards increased mortality in the ticagrelor arm [31, 32]. The primary end point in the 1814 patients in the US and Canada occurred in 11.9% of ticagrelor-treated patients compared with 9.6% of those on clopidogrel, although the difference was not statistically significant. In addition, an earlier phase 2 trial with ticagrelor conducted in America had shown unfavourable outcomes [33]. It has been suggested that this may be related to the fact that North American trial centres were subjected to monitoring by third party independent organisations whereas certain eastern European centres which delivered mortality reduction data for ticagrelor, were monitored by the trial sponsor. But the trial authors have asserted that after excluding the largest enrolling countries with results favouring ticagrelor, the overall result still favoured ticagrelor and that the paradox could indeed be related to a higher dose of aspirin used in North American centres [34]. Notably, the proportion of trial patients recruited from North American centres was small (<10%).

Ticagrelor was granted approval by US FDA in July 2011 and by NICE in Oct 2011 [35]. The European Society of Cardiology (ESC) recommendations on the use of the agents discussed above during myocardial revascularisation in unstable angina/NSTEMI and STEMI are shown in (Tables **1** and **2**) respectively [36].

### Cangrelor

Cangrelor is administered intravenously and like prasugrel and ticagrelor selectively blocks the platelet P2Y_12_ receptor. It is a non-thienopyridine unlike clopidogrel or prasugrel, with a rapid-onset and offset of action - plasma half-life of 3 to 6 minutes. Importantly, the profound and rapid antiplatelet effect of cangrelor is reversible in comparison with the irreversible blockade induced by clopidogrel or prasugrel. The platelet function normalises within 30 to 60 minutes after discontinuation of cangrelor infusion [37]. *In vitro* studies have demonstrated that cangrelor has additional antiplatelet effect when added to the platelets of patients receiving long-term treatment with clopidogrel [38, 39]. 

Two joint phase 3 studies [40, 41] conducted by the Cangrelor versus Standard Therapy to Achieve Optimal Management of Platelet Inhibition (CHAMPION) group of investigators reported negative results for cangrelor in the setting of PCI with or without ACS. The CHAMPION PLATFORM trial [40] compared cangrelor versus placebo and the CHAMPION PCI trial [41] cangrelor versus clopidogrel. Both the trials included patients with predominantly acute coronary syndromes for whom a strategy of deferred ADP-receptor blockade was chosen, i.e., after coronary anatomy was determined. The major difference between the two trials was the timing of the administration of clopidogrel – clopidogrel was loaded at the start of PCI in the CHAMPION PCI trial but was given towards or at the end of PCI in the CHAMPION PLATFORM study.

In the CHAMPION PLATFORM trial, 5362 patients with NSTEMI or unstable angina who had an angiographic lesion amenable to PCI, were randomised to receive either cangrelor or placebo infusion. There was no difference in the occurrence of the composite primary end point between the cangrelor and placebo arms; 7.0% versus 8.0%; (odds ratio [OR], 0.87; 95% CI, 0.71 to 1.07; P=0.17) [40].

In the CHAMPION PCI study patients (n=8877) with STEMI and stable angina were included in addition to those presenting with NSTEMI. Patients were randomly assigned to either cangrelor infusion or 600 mg clopidogrel following angiography. There was no difference in the incidence of the primary end point between the two groups at 48 hours; 7.5% versus 7.1% (OR, 1.05; 95% CI, 0.88 to 1.24; P=0.59) [41].

Bleeding risk was not increased with cangrelor as determined by the TIMI or Global Utilization of Streptokinase and Tissue Plasminogen Activator for Occluded Coronary Arteries (GUSTO) criteria. One of the main reasons for the negative results of these trials is thought to be the over-diagnosis of periprocedural myocardial infarction as part of the composite primary endpoint. The biomarker thresholds used to define MI were set quite low and events in the making could have been misinterpreted as being new, especially as the time-to-PCI was short. In addition, elevated biomarkers at baseline made diagnosis of new events difficult. Furthermore, use of 600 mg of clopidogrel rather than 300 mg could have increased antiplatelet efficacy in the control arm, thus making cangrelor appear less effective [42]. Failure of this trial made the future of cangrelor less bright.

Subsequently, as the antiplatelet effects of cangrelor were fully reversible, a role in patients awaiting CABG was envisaged and this hypothesis was tested in the Maintenance of Platelet Inhibition with Cangrelor after Discontinuation of Thienopyridines in Patients Undergoing Surgery (BRIDGE) study [43]. In this, 210 patients with ACS or coronary stents on ticlopidine, clopidogrel, or prasugrel awaiting CABG, were randomized to receive continuous infusions of cangrelor or placebo. Cangrelor was withdrawn one to six hours before CABG. Platelet function was monitored using the VerifyNow P2Y_12_ assay (Accumetrics). Significantly more patients on cangrelor than placebo had low levels of platelet reactivity throughout the entire infusion. There was no major difference in bleeding between the two groups. It must be noted that the BRIDGE study was powered for assessing platelet reactivity and safety and not hard clinical endpoints. Substituting cangrelor for oral thienopyridines prior to CABG in clinical practice requires further assessment in larger trials.

## PROTEASE-ACTIVATED RECEPTOR-1 (PAR-1) INHIBITORS

PAR-1 inhibitors are a new class of antiplatelet agents which cause platelet blockade *via* pathways that are different from that of aspirin, clopidogrel, prasugrel or ticagrelor. Thrombin-induced platelet aggregation is the main target of these agents. Vorapaxar and atopaxar are the PAR-1 inhibitors that have or are being evaluated in clinical studies. Thrombin, a serine protease is responsible for the generation of fibrin and in addition a potent agonist of platelets through its interaction with protease-activated receptors (PARs) [44]. Vorapaxar selectively and potently inhibits thrombin-induced platelet aggregation [45, 46]. Atopaxar has been shown to have synergistic effects with aspirin and a combination of aspirin and clopidogrel in human volunteers [47]. Phase 2 trials of vorapaxar [48, 49] and atopaxar [50, 51] had shown trends toward reductions in recurrent thrombotic events without increasing risk of intracranial bleeding.

Vorapaxar was subsequently evaluated in two large trials that were published this year – one in patients with acute coronary syndromes [52] and the other in patients with stable atherosclerotic disease [53]. Both trials demonstrated significantly increased bleeding risk with Vorapaxar.

The Thrombin Receptor Antagonist for Clinical Event Reduction in Acute Coronary Syndrome (TRACER) trial compared vorapaxar with placebo in nearly 13,000 patients presenting with NSTEMI ACS [52]. The study was terminated early after a safety review, owing to a significantly increased incidence of bleeding including intracranial haemorrhage in the vorapaxar arm. There was an absolute excess at two years of two moderate or severe bleeds, nearly one additional intracranial haemorrhage, and about five TIMI clinically relevant bleeds for every 100 patients treated. In addition, no reduction in ischaemic events was demonstrable with vorapaxar. 

In another trial, the Thrombin Receptor Antagonist in Secondary Prevention of Atherothrombotic Ischemic Events (TRA 2P)–Thrombolysis in Myocardial Infarction (TIMI) 50 trial, over 26,000 patients with a history of stable atherosclerosis were randomised to receive vorapaxar versus placebo [53]. At 3-years follow-up there was significant reduction in ischaemic events in the vorapaxar group compared to the placebo group (9.3% vs 10.5%, HR for the vorapaxar group, 0.87; 95% CI, 0.80 to 0.94; P<0.001). But this came at a cost of increased bleeding – GUSTO moderate or severe bleeding (HR 1.66; 95% CI 1.43 to 1.93; P<0.001), TIMI clinically significant bleeding (HR 1.46; 95% CI 1.36 to 1.57; P<0.001) and intracranial bleeds (HR 1.94; 95% CI 1.39 to 2.70; P<0.001) [53]. The future of this new class of antiplatelet agents is uncertain given the unfavourable results from these two large randomised studies.

## GLYCOPROTEIN IIB/IIIA INHIBITORS (GPI):

Glycoprotein (GP) IIb/IIIa receptor is the most abundant and only platelet-specific integrin receptor found on the surface of platelets. It transmits signals bidirectionally across the plasma membrane of platelets [54]. Fibrinogen, a plasma glycoprotein synthesised in the liver, is the receptor’s major ligand and its engagement to the receptor mediates formation of platelet aggregates which is central to thrombus formation. As the binding of the GP IIb/IIIa receptor to fibrinogen is the final common pathway in platelet aggregation, agents that block these receptors can impair platelet-dependent thrombogenesis irrespective of the metabolic pathway responsible for initiating the same. The feasibility of such blockade was demonstrated in the early 1980s by the development of peptides and antibodies that could interact with GPIIb/IIIa, thereby blocking ligand binding to the receptor and inhibiting platelet aggregation [55, 56]. GPI are currently approved as adjunctive therapy to reduce ischaemic complications of PCI and/or ACS. The first GPI to be tested, approved and used extensively in man, is the chimeric 7E3 Fab molecule designated abciximab (ReoPro [57]. This agent was a Fab fragment based on the monoclonal mouse antibody 7E3 which was humanised in order to reduce immunogenicity and prevent patients from raising a mouse-antibody-specific antibody response against non-human antibodies. Abciximab binds with the high-affinity GPI receptor, resulting in slow off-rate kinetics and long dissociation from platelet, despite the short plasma half-life of the drug. Thus, the platelet inhibition with abciximab lasts 48 hrs, but with the addition of ticlopidine the inhibitory effect of abciximab lasts several days after the drug is discontinued [57]. Three other GPI drugs were developed and were tested in trials. The first was Eptifibatide which is a cyclic peptide and a selective inhibitor with a short half-life. The second tirofiban, a small non-peptide antagonist, that causes rapid (5 min) and selective blockade of GP IIb/IIIa receptors. The antiplatelet effects of tirofiban are reversible in 4-6 hours. The third compound is lamifiban, a synthetic, non-peptide selective receptor blocker with a half-life of 4 hours. This review will focus only on abciximab, as this is the GPI that has been tested extensively and currently widely available. 

The initial abciximab trials, all of which were done in the setting of PCI, on top of aspirin and UFH, show a drug-specific effect; there was a more pronounced reduction in the endpoint of death or nonfatal MI at 30 days. Three-year follow-up of patients who entered the first abciximab trial (The Evaluation of 7E3 for the Prevention of Ischaemic Complications- EPIC study) indicates a 60% reduction in mortality; overall the mortality reduction at the latest point of follow-up in the abciximab trials was 35% [58-60]. The most impressive effect for mortality reduction is that reported in Evaluation of Platelet IIb/IIIa Inhibitor for Stenting (EPISTENT) trial, which included patients with both stable coronary disease and ACS who had coronary lesions that were suitable for stenting. One-year mortality amongst stented patient was reduced - RRR of 58% for mortality (2.4% for the placebo group vs 1.0% for the abciximab group) [61]. However, abciximab did not show any benefit in reduction of death or recurrent MI in ACS patients who were not scheduled to undergo early revascularisation in GUSTO IVACS trial [62].

The use of GPI in STEMI has also been shown to be beneficial in many trials when used as adjunctive therapy with both pharmacological and mechanical reperfusion [63, 64]. Most of these studies however, were performed in patients not pre-treated with high dose clopidogrel and in some, even without the routine use of stents [65, 66]. The optimal timing of abciximab use also remains controversial. Pooled data from three randomized placebo controlled trials showed that the use of GPI was associated with a 34% RRR of death or MI during 72 hours of medical management prior to PCI and an additional 41% RRR in the same endpoints in the 48 hours following PCI - when PCI was performed during administration of the study drug [67]. However, in patients with STEMI, upstream use of abciximab or a combination of reteplase and abciximab did not improve clinical outcomes at 90-days (including mortality) compared to the use of abciximab during and after primary PCI (PPCI). The bleeding risk was significantly higher in the combination upstream abciximab plus reteplase group (14.5%) and upstream abciximab group (10.1%) compared to PPCI group (6.9%) (P<0.001 for the comparison of combination-facilitated PCI with primary PCI) [68].

The Bavarian Reperfusion Alternatives Evaluation-3 (BRAVE-3) study tested whether abciximab provided additional benefit to high dose clopidogrel loading (600 mg) in patients with STEMI due to undergo PPCI. The trial showed no benefit of abciximab versus placebo in reducing infarct size. The study was not powered to evaluate reduction in mortality [69].

Recently, The Abciximab Intracoronary versus intravenously Drug Application in ST-Elevation Myocardial Infarction (AIDA-STEMI) study compared intracoronary versus intravenous bolus use of abciximab during PPCI in STEMI, with subsequent intravenous infusion for 12 hrs (n= 2065) [70]. The primary endpoint was a composite of all-cause mortality, recurrent infarction, or new congestive heart failure within 90 days of randomisation. Intracoronary, as compared with intravenous abciximab, resulted in a similar rate of the primary endpoint at 90 days (7.0% vs 7.6%; OR 0.91; 95% CI 0.64-1.28; P=0.58). The incidence of death (4.5% vs 3.6%; 1.24; 0.78-1.97; P=0.36) and reinfarction (1.8% vs 1.8%; 1.0; 0.51-1.96; P=0.99) did not differ between the treatment groups, whereas less patients in the intracoronary group had new congestive heart failure (2.4%vs 4.1%; 0.57; 0.33-0.97; P=0.04) [70].

The routine use of abciximab during PCI for ACS is not favoured in many centres, including ours, considering the results from the latest studies. The use of abciximab should be undertaken when the risk-benefit ratio suggests a potential benefit for the patient. Abciximab, however, still remains the adjunctive therapy of choice in some patients who have high intracoronary thrombus burden observed during PPCI. The high risk of bleeding noted with abciximab can be considerably reduced with a radial approach for PPCI.

## ANTI-THROMBIN AGENTS

Thrombin is a 308-amino acid protease that cleaves peptide bonds in selective substrates, including fibrinogen, factor V, factor VIII, and factor XIII [71]. It is a potent natural platelet activator that plays a pivotal role in the process of tissue injury, coagulation, and the platelet response [72]. During the primary phase of aggregation, platelets are loosely linked to each other by “fibrinogen bridges” between GP IIb/IIIa receptors. Thrombin is generated at the blood–plaque interface in association with the cellular membranes on cells and platelets and converts fibrinogen to fibrin, stabilizing the growing thrombus by cross-linking fibrin. 

### Bivalirudin

Bivalirudin, a direct thrombin inhibitor, binds specifically to thrombin at its active catalytic site and at the exosite-1 docking locus [73]. It competitively inhibits thrombin with high affinity and is a short-acting agent, with a half-life of 25 minutes [74, 75]. Bivalirudin has predictable linear pharmacokinetics and hence does not require laboratory monitoring of blood coagulation parameters.

The role of Bivalirudin in ACS has been studied in 2 large trials. The Acute Catheterization and Urgent Intervention Triage strategY (ACUITY) trial was a large-scale (n=13,819), prospective, multicenter, randomized study designed to determine the optimal anticoagulation regimen in patients with moderate- to high risk ACS being treated with an early invasive strategy [76]. In this trial, bivalirudin compared with a combination of heparin and GPI, resulted in a non-inferior rate of the composite ischaemic end point (7.8% and 7.3%, respectively; P=0.32) and a significantly reduced rate of major bleeding (3.0% and 5.7%; P<0.001; RR 0.53; 95% CI, 0.43 to 0.65). Bivalirudin monotherapy compared to heparin plus GPI reduced major bleeding in all prespecified subgroups which included patients who had positive or negative tests for biomarkers, those who did or did not undergo PCI, those who were randomly assigned to immediate or deferred treatment with GPI, and those who did or did not undergo early angiography [76].

The second major trial was the Harmonizing Outcomes with Revascularization and Stents in Acute Myocardial Infarction (HORIZONS-AMI), which was the first randomised study to evaluate the clinical value of Bivalirudin in patients with STEMI undergoing PPCI (n=3602) [77]. At 30 days, patients who received bivalirudin alone, as compared with those who received heparin in combination with a GPI had similar rates of MACE (5.4% and 5.5%, respectively; P=0.95). Patients in the bivalirudin arm, however, had a significantly lower rate of major bleeding when compared to patients in the GPI arm (4.9% vs. 8.3%; RR, 0.60; 95% CI, 0.46 to 0.77; P<0.001). There was a significantly higher rate of acute stent thrombosis (<24 hrs) in the bivalirudin arm (1.3% vs 0.3%, P<0.001). The stent thrombosis rate at 30 days, however, was not significantly different between the arms (1.2% vs 1.7%, P=0.28) [77]. Furthermore, at 3 years, patients allocated to bivalirudin monotherapy had lower rates of all-cause mortality (5·9% vs 7·7%, P=0·03), cardiac mortality (2·9% vs 5·1%, P=0·001), re-infarction (6·2%vs 8·2%, P=0·04), and non CABG major bleeding (6·9% vs 10·5%, P=0·0001) with no significant differences in ischaemia-driven target vessel revascularisation, stent thrombosis, or composite adverse events [78].

Considering the comparable efficacy to heparin plus GPI, lower bleeding rates and indeed cost effectiveness, bivalirudin is being increasingly used in ACS, particularly in PPCI for STEMI. The slightly increased risk of acute stent thrombosis in bivalirudin should be borne in mind and ideally patients should be kept in the PCI unit for the first 24 hrs after PPCI. 

### Factor Xa inhibitors- Fondaparinaux

Factor Xa inhibition following ACS blocks amplification of thrombin generation and subsequent clot formation, reducing risk of recurrent MI, stroke and death. Fondaparinux is a synthetic pentasaccharide that binds ATIII, resulting in a 340-fold increase in the rate of FXa inhibition over the basal rate [79]. The compound is administered subcutaneously, is rapidly absorbed and has linear pharmacokinetics. It results in effective anticoagulation with once-daily dosing with no apparent need for monitoring. 

Two large clinical trials have been published in which fondaparinux was evaluated in the setting of ACS. In the Fifth Organization to Assess Strategies in Acute Ischemic syndromes (OASIS-5) trial, 20,078 patients admitted with ACS were treated with either fondaparinux or enoxaparin [80]. Patients with STEMI were excluded from this study. The primary objective of the study was to establish non-inferiority of fondaparinux compared to enoxaparin with respect to death, MI, or refractory ischemia at 9 days. The main safety outcome was bleeding at 9 days. After 9 days of follow-up, fondaparinux met the non-inferiority criterion, with 5.8% of patients in either arm experiencing the primary end point. At 30 days, there was a trend favouring patients randomized to fondaparinux with respect to the combination of death, MI, and refractory ischemia. This trend was driven by a small but significant reduction in mortality among patients randomized to fondaparinux (2.9% vs 3.5%; P=0.02). These differences held up at 180 days, when mortality was lower among patients on fondaparinux than those treated with enoxaparin (5.8% vs 6.5%; P=0.05). Fondaparinux resulted in lower bleeding compared to enoxaparin at 9 days (2.2% vs 4.1%; P< 0.001), 30 days (3.1% vs 5%; P<0.001) , and at the end of the study (4.3%vs 5.8%; P< 0.001) [80].

In the subsequent OASIS-6 trial, 12 092 patients presenting with STEMI within 24 hours of symptom presentation were randomized to receive antithrombotic therapy with fondaparinux or to “usual care.” This trial was quite complex, because “usual care” meant either unfractionated heparin (UFH) or no antithrombotic therapy (placebo) [81]. The primary outcome measured was death or recurrent MI at 30 days. As with OASIS-5, bleeding complications were the main safety outcome. Fondaparinux resulted in a significant reduction in the rate of death or reinfarction at 9 days (7.4% vs 8.9%; P=0.003), 30 days (9.7% vs 11.2%; P=0.008) and at the end of study (13.4% vs 14.8%; P=0.008). There was no difference in bleeding complications between the two groups (1.0% vs 1.3%, P=0.13). However, when safety outcomes among patients undergoing PCI were analyzed in detail, it was noted that there was a trend toward more severe bleeding (16 vs 9 patients), as well as significantly more guiding catheter thrombosis (22 vs 0 patients; P<0.001), in the fondaparinux arm, compared with the UFH arm. Nevertheless, these events occurred in a small number of participants.. 

The results of these studies suggest that therapy with fondaparinux is less likely to be of benefit among ACS patients treated with an early invasive strategy. In all other patient groups studied, however, fondaparinux appears to offer a significant advantage over other anticoagulants, especially with regard to the risk of bleeding. 

### Oral Factor Xa Inhibitors

Rivaroxaban, the first oral factor Xa inhibitor does not require an antithrombin cofactor for its activity. Rivaroxaban was tested in a large phase 3 trial, the Anti-Xa Therapy to Lower Cardiovascular Events in Addition to Standard Therapy in Subjects with Acute Coronary Syndrome–Thrombolysis in Myocardial Infarction 51(ATLAS ACS 2-TIMI 51) as adjunctive therapy in patients with a recent ACS [82]. In this, 15,526 patients with a recent ACS were randomized to receive twice-daily doses of either 2.5 mg or 5 mg of rivaroxaban or placebo for up to 31 months (mean of 13 months). All patients were stabilised medically before enrolment and initial management strategies including revascularisation were completed. The median time from index event to randomisation was 4.7 days. Premature discontinuation of the drug was similar in all 3 groups ranging from 25-30%. Rivoraxaban significantly reduced the composite primary efficacy endpoint of death from cardiovascular causes, MI, or stroke, as compared with placebo (8.9% and 10.7% respectively, HR 0.84, CI 0.74-0.96, P=0.008). Therefore, the composite endpoint would be prevented in 1 patient if 56 patients were treated for 2 years with rivaroxaban. In addition, rivaroxaban reduced the risk of stent thrombosis when compared to placebo (2.3% vs 2.9%, HR 0.69, 95% CI 0.51-0.93, P=0.02). However non-CABG TIMI major bleeding was increased significantly in the rivaroxaban group compared to placebo at 2.1% and 0.6% respectively (HR 3.96, 95% CI 2.46-6.38, P<0.001). 

The composite primary end point benefit was noted in each of the doses of rivaroxaban when compared individually with placebo. However, there was no mortality benefit with the higher dose rivaroxaban (4.4% vs 4.5%, HR 0.95, 95% CI 0.76- 1.19, P=0.89), while the lower dose had significant mortality benefit (2.9% vs 4.5%, HR 0.68, 95% CI 0.53-0.87, P=0.004) when compared to placebo. The higher dose rivaroxaban group had higher all cause mortality and cardiovascular mortality when compared to low dose rivaroxaban (P= 0.009 for both comparisons). Also the non-CABG TIMI major bleeding was lower in patients receiving 2.5 mg dose than in the 5 mg dose patients (1.8% vs 2.4%, p 0.12), but when compared to placebo this was higher (1.8% vs 0.6%, p <0.001). 

The other oral Xa inhibitor is apixaban that was tested in Apixaban for Prevention of Acute Ischemic Events 2 (APPRAISE 2) trial, but was prematurely stopped because of higher bleeding risk in the apixaban group [83]. This trial showed that apixaban in addition to antiplatelet therapy in patients with ACS increased the number of major bleeding events without a significant reduction in ischemic endpoints. This had led to the ATLAS ACS trial investigators to exclude patients who had previous ischemic stroke or transient ischemic attack and were taking both aspirin and a thienopyridine.

The FDA rejected the use of rivaroxaban for ACS in May 2012, after extensive review and initial thoughts of approving the drug in a smaller dose. UK NICE is in the process of reviewing the use of rivaroxban in ACS and expected to decide in July 2013.

The European Society of Cardiology (ESC) recommendations on the use of the agents discussed above during myocardial revascularisation in unstable angina/NSTEMI and STEMI are shown in (Tables **1** and **2**) respectively [36].

## CONCLUSION

The last two decades have seen the advent of a motley array of antiplatelet agents. Some of these have demonstrated superiority of conventional therapies and found a place in routine clinical practice whereas others have failed to take flight. Following the sea change in antiplatelet treatment of ACS brought about by clopidogrel, newer P2Y_12_ agents such as prasugrel and ticagrelor have demonstrated clinical superiority, albeit at the cost of a slightly increased bleeding risk. Furthermore, these agents seem to be unable to overcome the Achille’s heel of irreversibility which poses hurdles especially when emergent CABG is needed or bleeding occurs. Cangrelor scores in certain areas with its rapid onset and offset of action and offers complete reversibility. But its biggest shortcoming is its failure to demonstrate superiority over clopidogrel in reducing incidence of ischaemic events. The new class of agents PAR-1 inhibitors showed initial clinical promise but phase 3 trials have demonstrated a significantly increased bleeding risk even in individuals with stable atherosclerotic disease. Their future therefore is shrouded in uncertainty. The GPI abciximab is useful in patients with a large intracoronary thrombus burden especially those undergoing PPCI. Bivalirudin, a thrombin inhibitor has shown superiority over a combination of heparin and GPI in patients with NSTEMI and STEMI undergoing PCI and its uptake has become more widespread lately. Subcutaneous factor Xa inhibitor fondaparinux offers benefit in patients with NSTEMI ACS in whom early invasive treatment is not planned. However the oral factor Xa inhibitors are not yet ready to be used in ACS. The options are therefore wide and potential combinations multitudinous. Clinicians must tailor the combination of antiplatelet and antithrombin strategies to suit individual patients’ risk profiles. Reduction of ischaemic burden must be balanced carefully against potential bleeding issues.

## Figures and Tables

**Table 1.** European Society of Cardiology (ESC) Guideline for the Use of Pharmacotherapy During Myocardial Revascularisation in Non ST Elevation Acute Coronary Syndrome (NSTEACS) which Includes NSTEMI and Unstable Angina [36]

NSTEMI		Class of Recommendation	Level of Evidence
**Antiplatelet therapy**			
	Aspirin	I	C
	Clopidogrel (with 600 mg loading as soon as possible)	I	C
	Clopidogrel (for 9-12 months afterPCI)	I	B
	Prasugrel	IIa	B
	Ticagrelor	I	B
	GPI (in patients with evidence of high intracoronary thrombus burden)		
	Abciximab (with DAPT)	I	B
	Tirofiban, eptifibatide	IIa	B
	Upstream GPI	III	B
**Anticoagulation**			
Very high risk of ischaemia	UFH (+GPI) or	I	C
	Bivalirudin monotherapy	I	B
Medium-high risk of ischaemia	UFH	I	C
	Bivalirudin	I	B
	Fondaparinux	I	B
	Enoxaparin	IIa	B
Low risk of ischaemia	Fondaparinux	I	B
	Enoxaparin	IIa	B

Adapted from 2010 ESC “Guidelines on myocardial revascularisation”: ASA- Aspirin, GPIIb-IIIa - Glycoprotein IIb/IIIa, DAPT- dual antiplatelet therapy, UFH- unfractionated heparin

**Table 2.** European Society of Cardiology (ESC) Guideline for the Use of Pharmacotherapy During Myocardial Revascularisation in ST Elevation Myocardial Infarction (STEMI) [36]

STEMI		Class of Recommendation	Level of Evidence
**Antiplatelet therapy**			
	Aspirin	I	B
	Clopidogrel (with 600 mg loading as soon as possible)	I	C
	Prasugrel	I	B
	Ticagrelor	I	B
	GPI (in patients with evidence of high intracoronary thrombus burden)		
	Abciximab (with DAPT)	IIa	A
	Tirofiban	IIa	B
	Eptifibatide	IIb	B
	Upstream GPI	III	B
**Anticoagulation**			
	Bivalirudin monotherapy	I	B
	UFH	I	C
	Fondaparinux	III	B

Adapted from 2010 ESC “Guidelines on myocardial revascularisation” ASA- Aspirin, GPIIb-IIIa - Glycoprotein IIb/IIIa, UFH- unfractionated heparin

## References

[1] Monroe D M, Hoffman M, Roberts H R (2002). Platelets and thrombin generation. Arterioscler Thromb Vasc Biol.

[2] Balsano F, Rizzon P, Violi F (1990). Antiplatelet treatment with ticlopidine in unstable angina. A controlled multicenter clinical trial. The Studio della Ticlopidina nell'Angina Instabile Group. Circulation.

[3] Rocca B, Patrono C (2005). Determinants of the interindividual variability in response to antiplatelet drugs. J Thromb Haemost.

[4] Jarvis B, Simpson K (2000). Clopidogrel: a review of its use in the prevention of atherothrombosis. Drugs.

[5] (1996). A randomised, blinded, trial of clopidogrel versus aspirin in patients at risk of ischaemic events (CAPRIE).CAPRIE Steering Committee. Lancet.

[6] Yusuf S, Zhao F, Mehta S R, Chrolavicius S, Tognoni G, Fox K (2001). Effects of clopidogrel in addition to aspirin in patients with acute coronary syndromes without ST-segment elevation. N Engl J Med.

[7] Mehta S R, Yusuf S, Peters R J (2001). Effects of pretreatment with clopidogrel and aspirin followed by long-term therapy in patients undergoing percutaneous coronary intervention: the PCI-CURE study. Lancet.

[8] Steinhubl S R, Berger P B, Mann J T (2002). Early and sustained dual oral antiplatelet therapy following percutaneous coronary intervention: a randomized controlled trial. JAMA.

[9] Chen Z M, Jiang L X, Chen Y P (2005). Addition of clopidogrel to aspirin in 45,852 patients with acute myocardial infarction: randomised placebo-controlled trial. Lancet.

[10] Sabatine M S, Cannon C P, Gibson C M (2005). Addition of clopidogrel to aspirin and fibrinolytic therapy for myocardial infarction with ST-segment elevation. N Engl J Med.

[11] Schomig A (2009). Ticagrelor--is there need for a new player in the antiplatelet-therapy field?. N Engl J Med.

[12] Serebruany V L, Steinhubl S R, Berger P B, Malinin A I, Bhatt D L, Topol E J (2005). Variability in platelet responsiveness to clopidogrel among 544 individuals. J Am Coll Cardiol.

[13] Ho P M, Maddox T M, Wang L (2009). Risk of adverse outcomes associated with concomitant use of clopidogrel and proton pump inhibitors following acute coronary syndrome. JAMA.

[14] Juurlink D N, Gomes T, Ko D T (2009). A population-based study of the drug interaction between proton pump inhibitors and clopidogrel. Cmaj.

[15] Gilard M, Arnaud B, Cornily J C (2008). Influence of omeprazole on the antiplatelet action of clopidogrel associated with aspirin: the randomized, double-blind OCLA (Omeprazole CLopidogrel Aspirin) study. J Am Coll Cardiol.

[16] Sibbing D, Morath T, Stegherr J (2009). Impact of proton pump inhibitors on the antiplatelet effects of clopidogrel. Thromb Haemost.

[17] Small D S, Farid N A, Payne C D (2008). Effects of the proton pump inhibitor lansoprazole on the pharmacokinetics and pharmacodynamics of prasugrel and clopidogrel. J Clin Pharmacol.

[18] Depta J P, Bhatt D L Omeprazole and clopidogrel: Should clinicians be worried?. Cleve Clin J Med.

[19] O'Donoghue M L, Braunwald E, Antman E M (2009). Pharmacodynamic effect and clinical efficacy of clopidogrel and prasugrel with or without a proton-pump inhibitor: an analysis of two randomised trials. Lancet.

[20] Bhatt D L, Cryer B L, Contant C F (2011). Clopidogrel with or without omeprazole in coronary artery disease. N Engl J Med.

[21] Interaction between clopidogrel and proton-pump inhibitors. CHMP updates warning for clopidogrel-containing medicines. 2010 [cited 2010 Mar 17; Available from: http://www.ema.europa.eu/humandocs/PDFs/EPAR/Plavix/17494810en.pdf.

[22] Jernberg T, Payne C D, Winters K J (2006). Prasugrel achieves greater inhibition of platelet aggregation and a lower rate of non-responders compared with clopidogrel in aspirin-treated patients with stable coronary artery disease. Eur Heart J.

[23] Wiviott S D, Trenk D, Frelinger A L (2007). Prasugrel compared with high loading- and maintenance-dose clopidogrel in patients with planned percutaneous coronary intervention: the Prasugrel in Comparison to Clopidogrel for Inhibition of Platelet Activation and Aggregation-Thrombolysis in Myocardial Infarction 44 trial. Circulation.

[24] Brandt J T, Payne C D, Wiviott S D (2007). A comparison of prasugrel and clopidogrel loading doses on platelet function: magnitude of platelet inhibition is related to active metabolite formation. Am Heart J.

[25] Wiviott S D, Braunwald E, McCabe C H (2007). Prasugrel versus clopidogrel in patients with acute coronary syndromes. N Engl J Med.

[26] Prasugrel for the treatment of acute coronary syndromes with percutaneous coronary intervention. [NICE guidance, UK] 2009 [cited 2009 NICE technology appraisal guidance 182; Available from: http://www.nice.org.uk/nicemedia/live/12324/45849/45849.pdf.

[27] Storey R F, Husted S, Harrington R A (2007). Inhibition of platelet aggregation by AZD6140, a reversible oral P2Y12 receptor antagonist, compared with clopidogrel in patients with acute coronary syndromes. J Am Coll Cardiol.

[28] Husted S, Emanuelsson H, Heptinstall S, Sandset P M, Wickens M, Peters G (2006). Pharmacodynamics, pharmacokinetics, and safety of the oral reversible P2Y12 antagonist AZD6140 with aspirin in patients with atherosclerosis: a double-blind comparison to clopidogrel with aspirin. Eur Heart J.

[29] Wallentin L, Becker R C, Budaj A (2009). Ticagrelor versus clopidogrel in patients with acute coronary syndromes. N Engl J Med.

[30] Boersma E, Harrington R A, Moliterno D J (2002). Platelet glycoprotein IIb/IIIa inhibitors in acute coronary syndromes: a meta-analysis of all major randomised clinical trials. Lancet.

[31] Ohman E M, Roe M T (2011). Explaining the unexpected: insights from
the PLATelet inhibition and clinical Outcomes (PLATO) trial
comparing ticagrelor and clopidogrel. Editorial on Serebruany
"Viewpoint: Paradoxical excess mortality in the PLATO trial
should be independently verified" (Thromb Haemost 2011; 105.5). Thromb Haemost.

[32] Serebruany V L (2011). Viewpoint: paradoxical excess mortality in the PLATO trial should be independently verified. Thromb Haemost.

[33] Cannon C P, Husted S, Harrington R A (2007). Safety, tolerability, and initial efficacy of AZD6140, the first reversible oral adenosine diphosphate receptor antagonist, compared with clopidogrel, in patients with non-ST-segment elevation acute coronary syndrome: primary results of the DISPERSE-2 trial. J Am Coll Cardiol.

[34] Wallentin L, Becker R C, James S K, Harrington R A (2011). The PLATO
trial reveals further opportunities to improve outcomes in patients
with acute coronary syndrome. Editorial on Serebruany. "Viewpoint:
Paradoxical excess mortality in the PLATO trial should be
independently verified" (Thromb Haemost 2011; 105.5). Thromb
Haemost.

[35] Ticagrelor for the treatment of acute coronary syndromes. [NICE
guidance, UK] Oct 2011 [cited 2011 Oct; NICE technology appraisal
guidance 236; Available from: http://www.nice.org.uk/
nicemedia/live/13588/56819/56819.pdf..

[36] Wijns W, Kolh P, Danchin N (2010). Guidelines on myocardial revascularization. Eur Heart J.

[37] Storey R F, Sanderson H M, White A E, May J A, Cameron K E, Heptinstall S (2000). The central role of the P(2T) receptor in amplification of human platelet activation, aggregation, secretion and procoagulant activity. Br J Haematol.

[38] Storey R F, Wilcox R G, Heptinstall S (2002). Comparison of the pharmacodynamic effects of the platelet ADP receptor antagonists clopidogrel and AR-C69931MX in patients with ischaemic heart disease. Platelets.

[39] Behan M W, Fox S C, Heptinstall S, Storey R F (2005). Inhibitory effects of P2Y12 receptor antagonists on TRAP-induced platelet aggregation, procoagulant activity, microparticle formation and intracellular calcium responses in patients with acute coronary syndromes. Platelets.

[40] Bhatt D L, Lincoff A M, Gibson C M (2009). Intravenous platelet blockade with cangrelor during PCI. N Engl J Med.

[41] Harrington R A, Stone G W, McNulty S (2009). Platelet inhibition with cangrelor in patients undergoing PCI. N Engl J Med.

[42] Kastrati A, Ndrepepa G (2009). Cangrelor - a champion lost in translation?. N Engl J Med.

[43] Angiolillo D J, Firstenberg M S, Price M J (2012). Bridging antiplatelet therapy with cangrelor in patients undergoing cardiac surgery: a randomized controlled trial. JAMA.

[44] Leger A J, Covic L, Kuliopulos A (2006). Protease-activated receptors in cardiovascular diseases. Circulation.

[45] Chackalamannil S (2006). Thrombin receptor (protease activated receptor-1) antagonists as potent antithrombotic agents with strong antiplatelet effects. J Med Chem.

[46] Chackalamannil S, Xia Y, Greenlee W J (2005). Discovery of potent orally active thrombin receptor (protease activated receptor 1) antagonists as novel antithrombotic agents. J Med Chem.

[47] Serebruany V L, Kogushi M, Dastros-Pitei D, Flather M, Bhatt D L (2009). The in-vitro effects of E5555, a protease-activated receptor (PAR)-1 antagonist, on platelet biomarkers in healthy volunteers and patients with coronary artery disease. Thromb Haemost.

[48] Becker R C, Moliterno D J, Jennings L K (2009). Safety and tolerability of SCH 530348 in patients undergoing non-urgent percutaneous coronary intervention: a randomised, double-blind, placebo-controlled phase II study. Lancet.

[49] Goto S, Yamaguchi T, Ikeda Y, Kato K, Yamaguchi H, Jensen P (2010). Safety and exploratory efficacy of the novel thrombin receptor (PAR-1) antagonist SCH530348 for non-ST-segment elevation acute coronary syndrome. J Atheroscler Thromb.

[50] O'Donoghue M L, Bhatt D L, Wiviott S D (2011). Safety and tolerability of atopaxar in the treatment of patients with acute coronary syndromes: the lessons from antagonizing the cellular effects of Thrombin-Acute Coronary Syndromes Trial. Circulation.

[51] Wiviott S D, Flather M D, O'Donoghue M L (2011). Randomized trial of atopaxar in the treatment of patients with coronary artery disease: the lessons from antagonizing the cellular effect of Thrombin-Coronary Artery Disease Trial. Circulation.

[52] Tricoci P, Huang Z, Held C (2012). Thrombin-receptor antagonist vorapaxar in acute coronary syndromes. N Engl J Med.

[53] Morrow D A, Braunwald E, Bonaca M P (2012). Vorapaxar in the secondary prevention of atherothrombotic events. N Engl J Med.

[54] Hynes R O (1987). Integrins: a family of cell surface receptors. Cell.

[55] Lefkovits J, Plow E F, Topol E J (1995). Platelet glycoprotein IIb/IIIa receptors in cardiovascular medicine. N Engl J Med.

[56] Chen P, Sun C, XLiu J N (2005). A novel anti-platelet monoclonal antibody (3C7) specific for the complex of integrin alpha IIb beta3 inhibits platelet aggregation and adhesion. J Biol Chem.

[57] Coller B S (2001). Anti-GPIIb/IIIa drugs: current strategies and future directions. Thromb Haemost.

[58] Topol E J, Ferguson J J, Weisman H F (1997). Long-term protection from myocardial ischemic events in a randomized trial of brief integrin beta3 blockade with percutaneous coronary intervention.EPIC Investigator Group. Evaluation of Platelet IIb/IIIa Inhibition for Prevention of Ischemic Complication. JAMA.

[59] Topol E J, Lincoff A M, Kereiakes D J (2002). l. Multi-year followup
of abciximab therapy in three randomized, placebo-controlled
trials of percutaneous coronary revascularization. Am J Med.

[60] Topol E J, Byzova T V, Plow E F (1999). Platelet GPIIb-IIIa blockers. Lancet.

[61] (1998). EPISTENT investigators. Randomised placebo-controlled and balloon-angioplasty-controlled trial to assess safety of coronary stenting with use of platelet glycoprotein-IIb/IIIa blockade. Lancet.

[62] Simoons M L (2001). Effect of glycoprotein IIb/IIIa receptor blocker abciximab on outcome in patients with acute coronary syndromes without early coronary revascularisation: the GUSTO IV-ACS randomised trial. Lancet.

[63] De Luca G, Suryapranata H, Stone G W (2005). Abciximab as adjunctive therapy to reperfusion in acute ST-segment elevation myocardial infarction: a meta-analysis of randomized trials. JAMA.

[64] Kandzari D E, Hasselblad V, Tcheng J E (2004). Improved clinical outcomes with abciximab therapy in acute myocardial infarction: a systematic overview of randomized clinical trials. Am Heart J.

[65] Brener S J, Barr L A, Burchenal J E (1998). Randomized, placebo-controlled trial of platelet glycoprotein IIb/IIIa blockade with primary angioplasty for acute myocardial infarction.ReoPro and Primary PTCA Organization and Randomized Trial (RAPPORT) Investigators. Circulation.

[66] Montalescot G, Barragan P, Wittenberg O (2001). Platelet glycoprotein IIb/IIIa inhibition with coronary stenting for acute myocardial infarction. N Engl J Med.

[67] Boersma E, Akkerhuis K M, Theroux P, Califf R M, Topol E J, Simoons M L (1999). Platelet glycoprotein IIb/IIIa receptor inhibition in non-ST-elevation acute coronary syndromes: early benefit during medical treatment only, with additional protection during percutaneous coronary intervention. Circulation.

[68] Ellis S G, Tendera M, de Belder M A (2008). Facilitated PCI in patients with ST-elevation myocardial infarction. N Engl J Med.

[69] Mehilli J, Kastrati A, Schulz S (2009). Abciximab in patients with acute ST-segment-elevation myocardial infarction undergoing primary percutaneous coronary intervention after clopidogrel loading: a randomized double-blind trial. Circulation.

[70] Thiele H, Wohrle J, Hambrecht R (2012). Intracoronary versus intravenous bolus abciximab during primary percutaneous coronary intervention in patients with acute ST-elevation myocardial infarction: a randomised trial. Lancet.

[71] Becker R C (2005). Understanding the dynamics of thrombin in cardiovascular disease: pathobiology and biochemistry for the clinician. Am Heart J.

[72] Ashby CR D J, Kunapuli SP, Smith JB, Colman RW (2001). Platelet stimulatory and inhibitory receptors. Hemostasis and Thrombosis.

[73] Weitz J I, Bates E R (2003). Direct thrombin inhibitors in cardiac disease. Cardiovasc Toxicol.

[74] Eikelboom J, White H, Yusuf S (2003). The evolving role of direct thrombin inhibitors in acute coronary syndromes. J Am Coll Cardiol.

[75] Maraganore J M, Adelman B A (1996). Hirulog: a direct thrombin inhibitor for management of acute coronary syndromes. Coron Artery Dis.

[76] Stone G W, McLaurin B T, Cox D A (2006). Bivalirudin for patients with acute coronary syndromes. N Engl J Med.

[77] Stone G W, Witzenbichler B, Guagliumi G (2008). Bivalirudin during primary PCI in acute myocardial infarction. N Engl J Med.

[78] Stone G W, Witzenbichler B, Guagliumi G (2011). Heparin plus a glycoprotein IIb/IIIa inhibitor versus bivalirudin monotherapy and paclitaxel-eluting stents versus bare-metal stents in acute myocardial infarction (HORIZONS-AMI): final 3-year results from a multicentre, randomised controlled trial. Lancet.

[79] Alban S (2005). From heparins to factor Xa inhibitors and beyond. Eur J Clin Invest.

[80] Yusuf S, Mehta S R, Chrolavicius S (2006). Comparison of fondaparinux and enoxaparin in acute coronary syndromes. N Engl J Med.

[81] Yusuf S, Mehta S R, Chrolavicius S (2006). Effects of fondaparinux on mortality and reinfarction in patients with acute ST-segment elevation myocardial infarction: the OASIS-6 randomized trial. JAMA.

[82] Mega JL, Braunwald E, Wiviott SD (2012). Rivaroxaban in patients with a recent acute coronary syndrome. N Engl J Med.

[83] Alexander JH, Lopez RD, James S (2011). Apixaban with antiplatelet therapy after acute coronary syndrome. N Engl J Med.

